# Lysophosphatidic acid exerts protective effects on HEI-OC1 cells against cytotoxicity of cisplatin by decreasing apoptosis, excessive autophagy, and accumulation of ROS

**DOI:** 10.1038/s41420-023-01706-5

**Published:** 2023-11-15

**Authors:** Xiaogang An, Cuiping Zhong, Bang Han, Erfang Chen, Qingwen Zhu, Yang Yang, Rui Li, Runqin Yang, Dingjun Zha, Yu Han

**Affiliations:** 1 Department of Otolaryngology, Xijing Hospital, Air Force Medical University, Xi’an, 710032 Shaanxi Province China; 2 National Clinical Research Center for Otolaryngologic Diseases of Shaanxi sub center, Xi’an, 710032 Shaanxi Province China; 3 The 940th Hospital of Joint Logistics Support Force of People’s Liberation Army, Lanzhou, 730050 Gansu Province China

**Keywords:** Apoptosis, Experimental models of disease

## Abstract

Lysophosphatidic acid (LPA) is an active phospholipid signaling molecule that binds to six specific G protein-coupled receptors (LPA_1-6_) on the cell surface and exerts a variety of biological functions, including cell migration and proliferation, morphological changes, and anti-apoptosis. The earliest study from our group demonstrated that LPA treatment could restore cochlear F-actin depolymerization induced by noise exposure, reduce hair cell death, and thus protect hearing. However, whether LPA could protect against cisplatin-induced ototoxicity and which receptors play the major role remain unclear. To this end, we integrated the HEI-OC1 mouse cochlear hair cell line and zebrafish model, and found that cisplatin exposure induced a large amount of reactive oxygen species accumulation in HEI-OC1 cells, accompanied by mitochondrial damage, leading to apoptosis and autophagy. LPA treatment significantly attenuated autophagy and apoptosis in HEI-OC1 cells after cisplatin exposure. Further investigation revealed that all LPA receptors except LPA_3_ were expressed in HEI-OC1 cells, and the mRNA expression level of LPA_1_ receptor was significantly higher than that of other receptors. When LPA_1_ receptor was silenced, the protective effect of LPA was reduced and the proportion of apoptosis cells was increased, indicating that LPA-LPA_1_ plays an important role in protecting HEI-OC1 cells from cisplatin-induced apoptosis. In addition, the behavioral trajectory and in vivo fluorescence imaging results showed that cisplatin exposure caused zebrafish to move more actively, and the movement speed and distance were higher than those of the control and LPA groups, while LPA treatment reduced the movement behavior. Cisplatin caused hair cell death and loss in zebrafish lateral line, and LPA treatment significantly protected against hair cell death and loss. LPA has a protective effect on hair cells in vitro and in vivo against the cytotoxicity of cisplatin, and its mechanism may be related to reducing apoptosis, excessive autophagy and ROS accumulation.

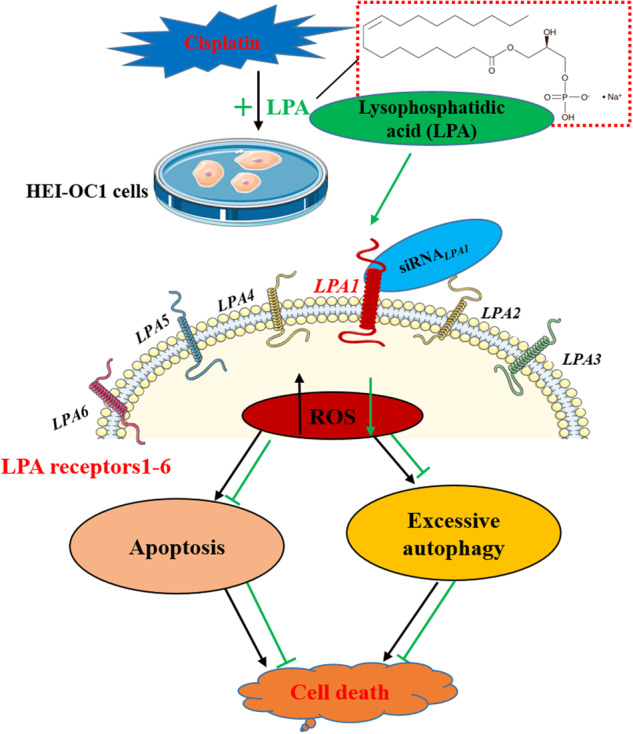

## Introduction

Hearing loss, a globally prevalent sensory disability, mainly occurs because of aging, medication use, or excessive noise exposure [1–4]. The platinum-based agent cisplatin is commonly recommended for treating solid tumors in humans, for example, melanoma, head and neck, lung, bladder, and ovarian tumors [5–7]. To date, ~2 billion dollars worth of platinum-based anticancer drugs have been marketed worldwide, and almost 50% of cancer patients are administered cisplatin for treatment [8]. However, Cisplatin is an ototoxic drug that can cause hearing loss [9]. A high incidence of severe ototoxicity is associated with cisplatin, which triggers hair cell death and subsequently results in loss of hearing [10, 11]. Cisplatin ototoxicity limits its therapeutic effects in patients with cancer, as approximately 80% of cisplatin-treated patients have at least 20 dB hearing loss [12]. Moreover, although several research studies are being conducted on cisplatin-induced ototoxicity, there are no available licensed drugs to prevent ototoxicity [13, 14]. Therefore, new agents or effective approaches are urgently required to prevent and treat cisplatin-induced ototoxicity.

Cisplatin induces apoptosis of cancer cells by inducing DNA damage, preventing DNA synthesis, and activating several transduction pathways [15, 16]. Ototoxicity induced by cisplatin remains to be fully characterized, and this effect may be mediated by several factors [17]. Cisplatin-induced ototoxicity promotes reactive oxygen species (ROS) accumulation, mitochondrial dysfunction, apoptosis, and autophagy [18, 19]. Hazlitt et al. reported that this ototoxicity might be due to the production of toxic ROS levels and cellular inflammatory responses toward apoptosis [20]. Inflammatory responses, combined with oxidative stress, lead to apoptosis and necrosis at the base and middle of the cochlea [21]. Moreover, ROS-induced cytotoxicity causes mitochondrial damage and reduces mitochondrial membrane potential, resulting in mitochondrial apoptosis and death [22]. Although autophagy activation is beneficial in some pathological states such as metabolic and neurodegenerative diseases, an excessive level of activation results in apoptotic cell death and promotes hepatic fibrosis development [4, 23]. Therefore, it remains controversial whether autophagy activation promotes or reduces cell survival.

The active phospholipid signaling molecule LPA (lysophosphatidic acid) exerts various biological effects such as cell migration and proliferation, morphological alterations, and antiapoptotic effects [24]. LPA binds to specific cell surface receptors (LPA_1–6_) to exert its influence. There are two subfamilies of LPA receptors: LPA_1-3_, which are G-protein-coupled (EDG) receptors; LPA_4-6_, which are non-EDG receptors [25]. LPA receptors are expressed in varying spatiotemporal patterns from embryonic development to adulthood, and LPA may activate different receptor isoforms to exert a variety of biological effects [26, 27]. LPA induces a motile and proinflammatory microglial phenotype via LPA_5_/protein kinase D-dependent pathways [28]. Selective LPA_1_ and LPA_3_ antagonists can inhibit the change in shape and aggregation of human platelets induced by LPA, thus indicating the regulatory role of LPA_1/3_ [29]. LPA’s effects on inner ear auditory cells are rarely studied, and the mechanisms underlying LPA’s effects on auditory cells remain unclear. At present, some compounds used to treat or prevent cisplatin-induced ototoxicity, including anti-inflammatory compounds (flunarizine and etanercept) and antioxidants (dexamethasone and sodium thiosulfate), are currently in clinical trials [30]. Based on our earlier findings on the efficacy of LPA in preventing noise-induced deafness [31], we speculate that LPA may prevent cisplatin ototoxicity. We also suggest that cochlea cell survival following cisplatin treatment may be influenced by LPA through binding to specific receptors (LPA_1–6_). In the present study, we validated these hypotheses by analyzing how LPA prevents the death of HEI-OC1 cells due to cisplatin exposure and the underlying mechanisms of action; we evaluated how cisplatin and LPA exposure influence the motor behavior in a zebrafish model by using a behavioral trajectory tracking system (EthoVision XT 17.0). We preliminarily investigated the influence of LPA_1-6_ expression and LPA_1_ silencing on the protective effect of LPA. These findings could enable to clarify the synergistic mechanism of LPA and its receptor.

## Results

### LPA protects HEI-OC1 cells against cytotoxicity of cisplatin

**Fig. 1:**
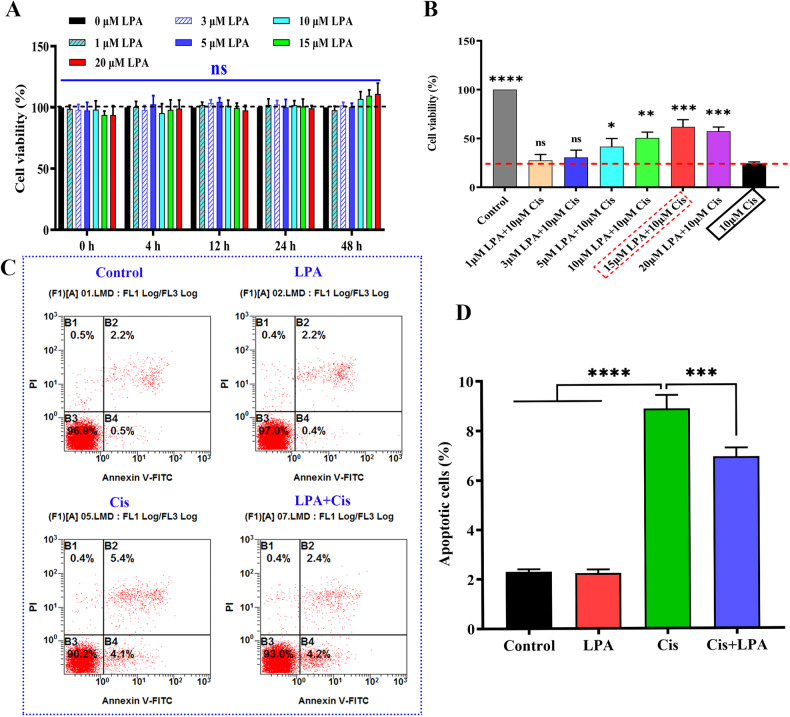
LPA protects HEI-OC1 cells against cytotoxicity of cisplatin. **A** HEI-OC1 cells were treated with increasing concentrations of LPA: 0 μM (vehicle control), 1, 3, 5, 10, 15, and 20 µM, and incubated for 0, 4, 12, 24, and 48 h. Cell viability was assessed by cell counting kit-8 (*n* = 6, Data are represented as means ± SD, *p < 0.05). **B** HEI-OC1 cells were exposed to LPA (1, 3, 5, 10, 15, and 20 µM) for 4 h and treated with cisplatin (10 µM) for 24 h. (*n* = 6, Data are represented as means ± SD. *p < 0.05 versus cisplatin group; **p < 0.01; ***p < 0.001; ns, non-significance). **C** Flow cytometric analysis of apoptotic cells in various treatment groups. Viable cells are shown by the Annexin V-negative and propidium iodide-negative quadrant (Q3, lower left quadrant). The Annexin V-positive and PI-negative quadrant (Q4, lower right quadrant) shows early apoptotic cells. The Annexin V-positive and PI-positive quadrant (Q2, upper right quadrant) shows late apoptotic and necrotic cells. The total number of apoptotic cells was the sum of early (Q4) and late (Q2) apoptotic cells (i.e., Q4 + Q2). *n* = 3, Data are represented as means ± SD. *p < 0.05 versus control group or LPA group. **D** Data shown in **C** are quantified. ****p < 0.0001 and *p < 0.05.

LPA pretreatment protects mice from noise-induced hearing loss (NIHL), thus suggesting that LPA is a potential drug to prevent hearing loss [31]. Here, we used HEI-OC1 cells to determine whether LPA shows preventive and protective effects on hearing loss due to cisplatin. First, we tested the viability of HEI-OC1 cells by using the CCK-8 kit (GlpBio Technology USA, USA, GK10001) to estimate LPA’s optimum concentration as well as pretreatment time. Various LPA concentrations (0, 1, 3, 5, 10, 15, and 20 µM, *n* = 6) were used for treating the cells for varying time points (i.e., 0, 4, 12, 24, and 48 h). Figure 1A shows that different concentrations (1–20 uM) of LPA-treated HEI-OC1 cells for 0–48 h. There was no significant difference in cell viability between groups, indicating that LPA is not toxic to cells and the length of incubation. Thus, the pretreatment time of LPA in our subsequent study was chosen to be 4 h.

To determine the effect and optimal concentration of LPA to protect cells from cisplatin damage. Cells were pretreated with different concentrations of LPA (1, 3, 5, 10, 15, and 20 μ M) for 4 h and then treated with 10 μM cisplatin for 24 h. Cell viability assays were performed. The CCK-8 assay demonstrated that the cell viability percentage was 100%, 27.56 ± 6.65%, 30.55% ± 8.05%, 41.63% ± 8.99%, 50.51% ± 6.74%, 61.82% ± 8.45%, and 57.44% ± 4.61% for the control, 1, 3, 5, 10, 15, and 20 µM LPA groups, respectively, and 24.59 ± 1.58% for cisplatin-treated cells (Fig. 1B). The results showed that pretreatment of HEI-OC1 cells with 3–20 μM of LPA significantly increased cell viability (*p < 0.05 versus cisplatin group) and the protective effect showed a dose-dependence. In all, 15 μM of LPA showed a relatively better protective effect, and therefore, subsequent immunofluorescence, Immunoblot detection, Therefore, 15 μM LPA was used to treat the cells in subsequent immunofluorescence, immunoblot detection, flow cytometry, and other experiments.

### LPA alleviates apoptosis of HEI-OC1 cells caused by cisplatin

For validating and evaluating LPA’s effect on cell damage caused by cisplatin, TUNEL assay, and FCM were conducted to assess apoptotic cells. In FCM analysis, PI and Annexin V were used to mark dead cells as well as cells undergoing apoptosis, respectively. First, experimental samples were divided into the following four groups: PBS, 10 µM cisplatin, 15 µM LPA, and 10 µM cisplatin + 15 µM LPA. The apoptotic cell percentages in the cisplatin and LPA + cisplatin groups were 8.87% ± 0.55% and 6.93% ± 0.35% (Fig. 1C, D). Figure 1C, D data indicated that remarkable reduction in the apoptotic cell percentage by LPA pretreatment before cisplatin exposure (****p < 0.0001 and *p < 0.05, *n* = 3, means ± SD).

**Fig. 2:**
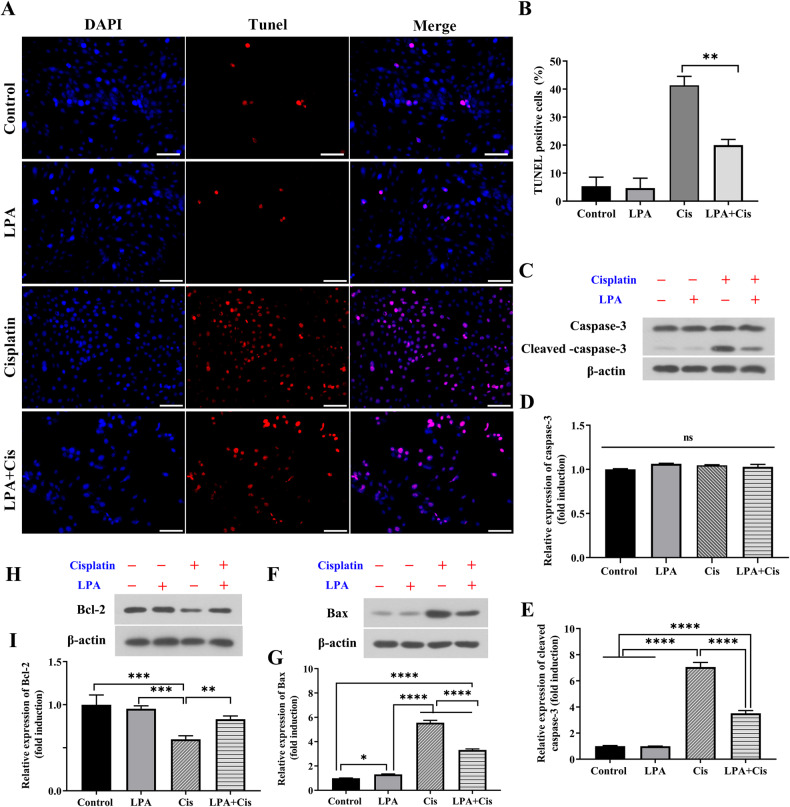
LPA protects cells by inhibiting cisplatin-induced activation of the apoptotic pathway. **A** Immunofluorescence staining with TUNEL and DAPI in the HEI-OC1 cells after different treatments, Scale bar = 100 um, *n* = 3. **B** Data shown in **A** were quantified (**p < 0.01). **C** Immunoblot detection of caspase 3/cleaved caspase 3 and β-actin expression in different treatment groups cells. **D** and **E** data shown in **C** were quantified (****p < 0.0001; ns, non-significance; *n* = 3). **F** Immunoblot detection of Bax and β-actin expression in different groups of HEI-OC1 cells. **G** Data shown in **F** were quantified. **H** WB assay of Bcl-2 and β-actin expression in different groups of HEI-OC1 cells. **I** Data shown in **H** were quantified (****p < 0.0001, **p < 0.01 and *p < 0.05, *n* = 3).

To further validate that LPA attenuated cisplatin-induced apoptosis in HEI-OC1 cells. We used the TUNEL assay to detect the number of apoptotic cells upon cisplatin exposure and LPA pretreatment. As shown in Fig. 2A, B, apoptotic cells were significantly increased in the cisplatin-exposed group, and LPA pretreatment significantly alleviated cisplatin-induced apoptosis. Immunoblot detection was conducted to evaluate expression of apoptosis-related proteins. As show in Fig. 2C–H, cleaved caspase-3 and Bax expression levels were significantly increased in HEI-OC1 cells after exposure to cisplatin when compared with those in the control and LPA groups (****p < 0.0001 and *p < 0.05, *n* = 3). Bax and cleaved caspase-3 were significantly downregulated after LPA (15 µM) treatment in comparison to those in the cisplatin group. The expression of Bcl-2 expression was significantly upregulated. The above results suggest that LPA treatment significantly reduces cisplatin-induced apoptosis in HEI-OC1 cells.

### Cisplatin-induced ROS production and cell apoptosis are inhibited by pretreatment with LPA

**Fig. 3:**
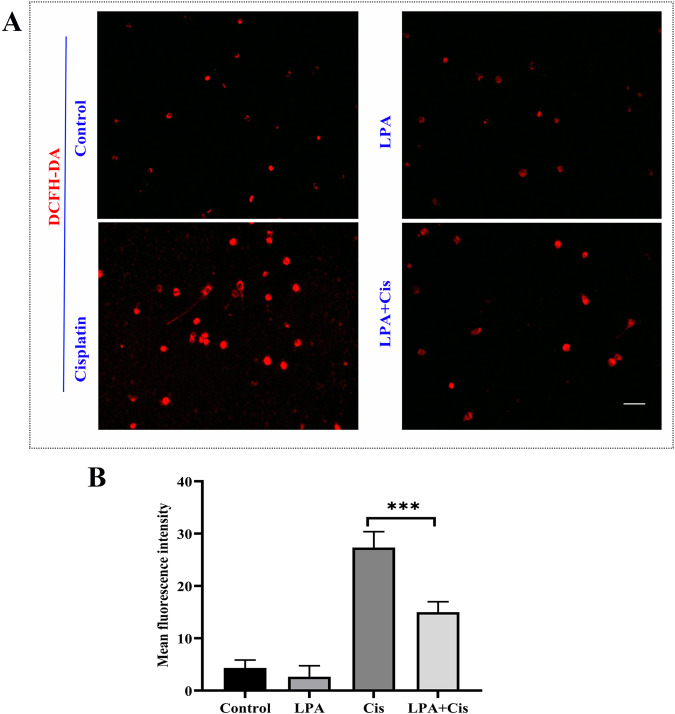
HEI-OC1 pretreatment with LPA inhibits ROS production and apoptosis induced by cisplatin. **A** DCFH-DA assay to detect intracellular ROS. HEI-OC1 cells were exposed to 0 µM cisplatin (vehicle control), 15 µM LPA, 15 µM LPA + 10 µM cisplatin, and 10 µM cisplatin. After 4-h LPA pretreatment, cisplatin was used to treat the cells for 24 h. **B** Data shown in **A** were quantified. ***p < 0.001, Scale bar = 100 μm.

Cisplatin treatment can increase ROS levels in cells [32–36], thereby leading to cell apoptosis and death. Intracellular ROS were detected using a DCFH-DA fluorescent probe [37] in HEI-OC1 cells of different groups (Fig. 3A, B). Thus, LPA pretreatment reduced ROS levels in HEI-OC1 cells, which were increased by cisplatin.

### Pretreatment with LPA inhibits cisplatin-induced ultrastructural changes in cells

**Fig. 4:**
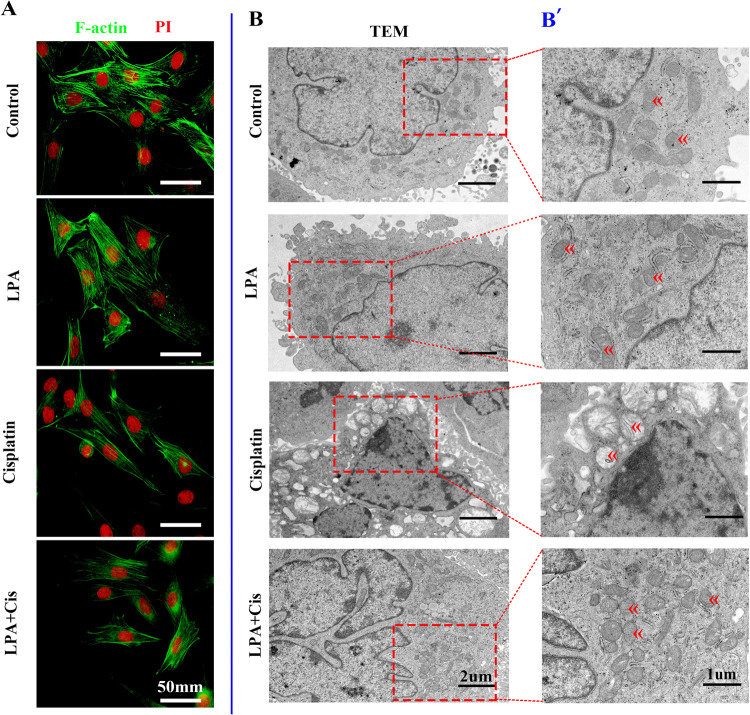
Pretreatment with LPA reduces cisplatin-induced ultrastructural changes in cells. **A** Morphological and structural changes in the different treatment groups. Scale bar: 50 mm. **B** Transmission electron microscopy study for the presence of HEI-OC-1 cells that underwent apoptosis. Cells were exposed to 0 µM cisplatin (vehicle control), 15 µM LPA, 15 µM LPA + 10 µM cisplatin, and 10 µM cisplatin. After 4-h LPA pretreatment, cells were subjected to cisplatin treatment for 24 h. **B**′ is a locally enlarged picture of the site shown by the red dashed box in each treatment group of Fig. **B**, mark the mitochondria with red arrows.

F/G-actin is an important protein for maintaining the cytoskeleton structure. We previously reported that long-term exposure to noise led to hearing loss and caused a change in the F/G-actin ratio, which may be related to damage to the cytoskeleton structure [31]. Here, we studied alterations in F-actin caused by cisplatin and LPA treatment. The control and LPA group cells showed normal morphology, and F-actin was arranged in a proper order and loosely distributed within the cell body. In contrast, cells in the cisplatin-treated group became slender and spindle-shaped, and F-actin in the cells was decreased and tightly arranged (Fig. 4A). LPA pretreatment remarkably reduced the ultrastructural changes in cells caused by cisplatin. Thus, LPA reduced cytoskeletal rearrangement induced by cisplatin.

We also examined the morphology of mitochondria and apoptotic bodies by TEM. We observed that exposure to cisplatin led to mitochondrial shrinkage and loss of mitochondrial crest (Fig. 4B). However, mitochondrial ultrastructure was maintained in the LPA-treated group. Thus, LPA attenuated the mitochondrial damage of HEI-OC1 cells due to cisplatin. Because cisplatin-treated cells showed several apoptotic bodies, cisplatin exposure may activate apoptotic pathways.

### HEI-OC1 cell treatment with LPA inhibits autophagy due to cisplatin

**Fig. 5:**
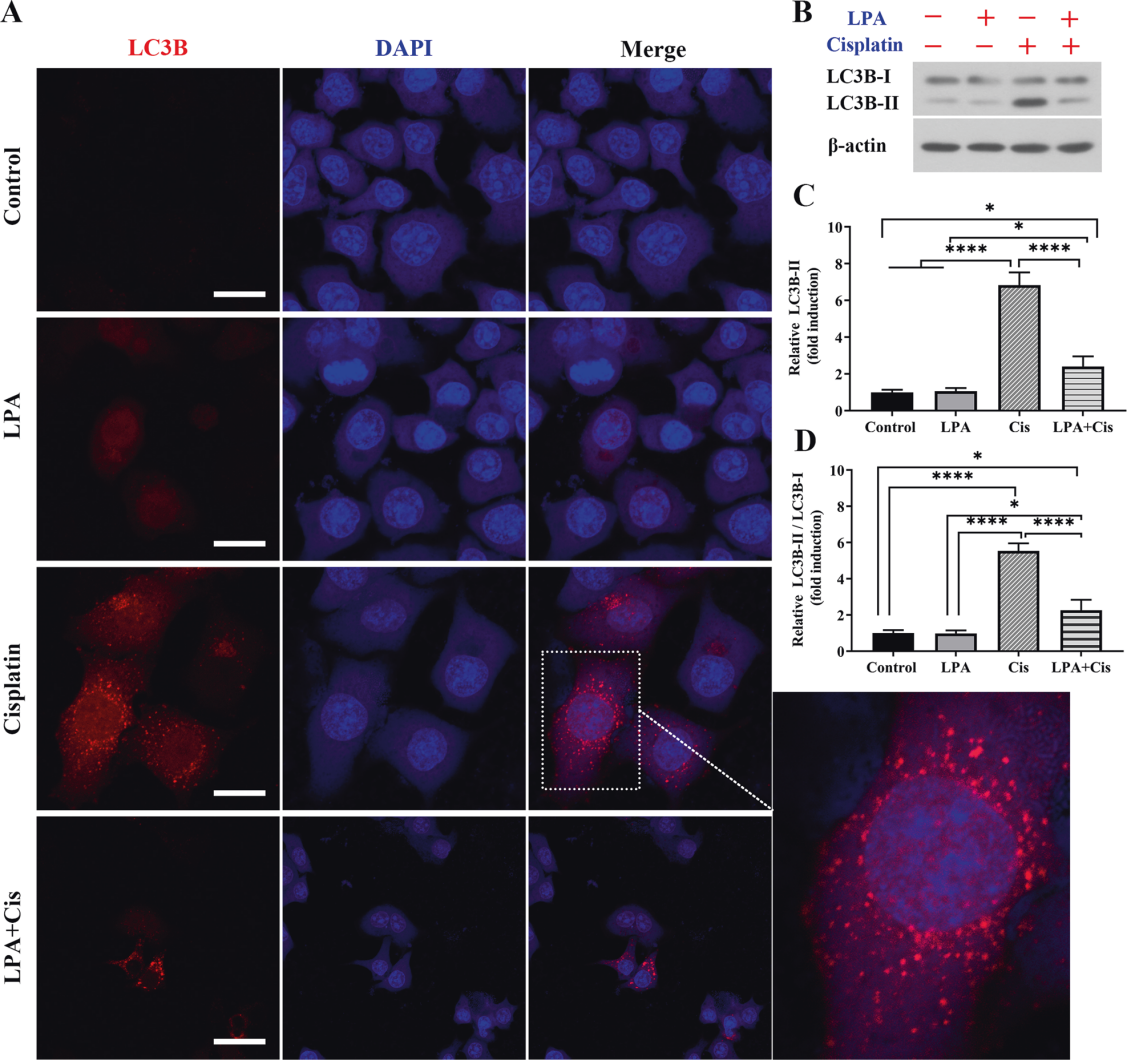
LPA treatment inhibited cisplatin-induced autophagy. **A** Immunofluorescence staining images of LC3B in HEI-OC1 cells exposed to cisplatin (10 µM) for 24 h and with or without 15 µM LPA. **B** Immunoblot detection of LC3B-II and LC3B-I expression in cisplatin- and/or LPA-exposed cells. **C**, **D** LC3B expression quantification in **B** ****p < 0.0001, *p < 0.05, Scale bar = 50 mm.

We assessed how LPA is involved in regulating autophagy in HEI-OC1 cells. Expression and distribution of the autophagy-related protein LC3B (Fig. 5A–D) in treated cells were determined by immunofluorescence (Fig. 5A). LPA treatment markedly inhibited the upregulation of cisplatin-induced autophagy, thus showing that LPA inhibited autophagy induced by cisplatin.

### Pretreatment with LPA treatment alleviates cisplatin-induced ototoxicity in zebrafish

**Fig. 6:**
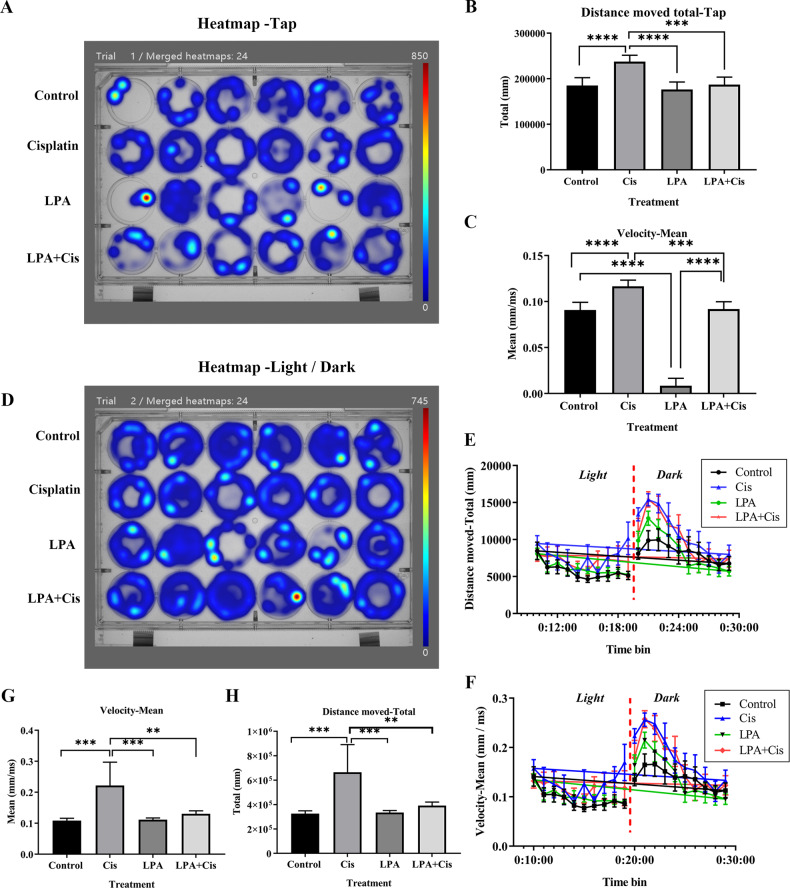
Tracking paths for the tap and light/dark photoperiodic variation model. **A**, **D** Heatmap of the tracked path of tap and light/dark modes for each experimental group containing six zebrafish individuals. Blue marks show the area of the passage of zebrafish, and the color intensity represents the tracked path’s cumulative frequency [77]. **B** Quantification of the running distance of zebrafish in each treatment group. **C** Quantification of the movement velocity of zebrafish in each treatment group. **E**, **F** Running distance and movement velocity analysis of zebrafish in each treatment group under light/dark conditions, respectively (light and dark cycles were run for 10 min each). **G** Quantification of the movement velocity of zebrafish in each treatment group. **H** Quantification of the running distance of zebrafish in each treatment group. Six zebrafish were studied in each group. *p* < 0.0001, ***p < 0.001, **p < 0.01 and *p < 0.05, *n* = 6.

**Fig. 7:**
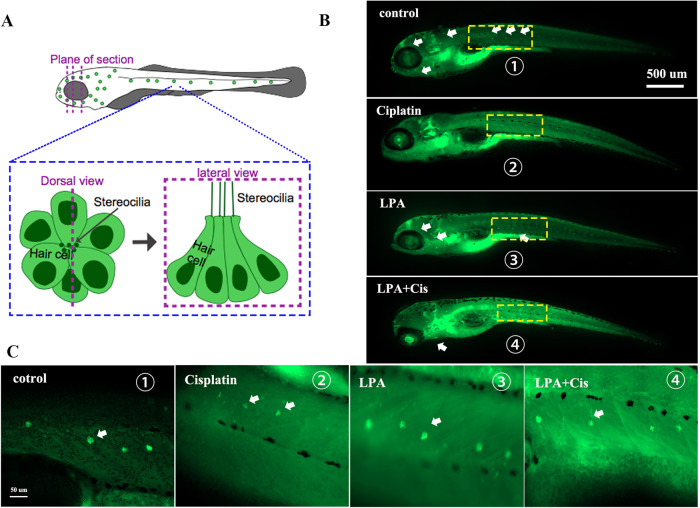
LPA protects against cisplatin-induced HCs loss in zebrafish lateral lines in vivo. **A** Schematic diagram of the distribution of hair cells in the lateral line neuromast throughout the body of zebrafish and the observation of the dorsal and lateral view of hair cells. **B** Survival of neuromast hair cells in the lateral line of zebrafish in different treatment groups. **C** A partial enlarged view of the box in Figure **B**. **Zebrafish strains:** [Et(krt4: EGFP)]; control group, do not process; Cisplatin group, 10 μM Cisplatin ; LPA group, 15 μM LPA; LPA+Cis group, adding 15 μM LPA for 4 h, then adding 10 μM Cisplatin for 24 h; The above groups were treated for the same time.

Zebrafish is an important model to test compounds that protect against toxicity caused by cisplatin in vivo; this is because their lateral line system has hair cells identical to those found in the inner ear of mammals. These cells can be readily reached through in vivo administration of drugs [38–40]. Therefore, we investigated the effects of cisplatin and LPA alone and in combination on Zebrafish embryonic development (10–96 hpf) and zebrafish larvae on day 5 following fertilization. The LPA and LPA + cisplatin groups were pretreated for 4 h with LPA (15 µM) and subjected to 24-h treatment with cisplatin (10 µM). A behavioral analysis system [41] was used to record and analyze the larvae’s locomotion parameters. Figure 6A–F shows that when compared with the other treatment groups, regardless of the tapping stimulation or light/dark cycle changes, cisplatin exposure caused zebrafish to move more actively, and their movement speed and distance were quite higher as compared to those of the control and LPA groups. Neuromast cell vitality was evaluated by Zebrafish [Et (krt4: EGFP)] labeled with lateral neuromast hair cells, cisplatin causes partial hair cell death and loss, and LPA treatment can significantly reduce hair cell death and loss, and has a significant protective effect (Fig. 7).

### Silencing of the LPA_1_ receptor blocks protective effects of LPA on HEI-OC1 cells from apoptosis due to cisplatin

**Fig. 8:**
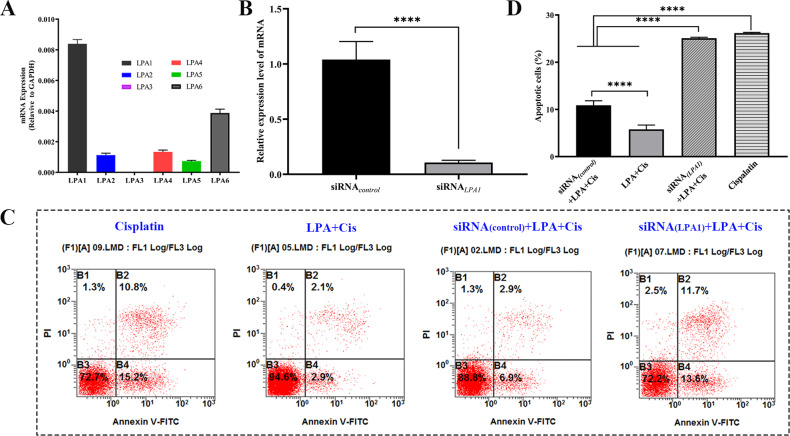
Silencing of the LPA_1_ receptor prevents LPA’s protective effects on cisplatin-induced apoptosis. **A** qRT-PCR for the relative expression of the LPA receptors _1–6_ (*n* = 3). **B** Effective silencing of the LPA_1_ receptor using siRNA. siRNA_(*LPA1*)_ expression was significantly decreased in HEI-OC1 cells as compared to that in cells transfected with siRNA_(control)_ ****p < 0.0001, *n* = 3, as estimated by one-way ANOVA. **C** FCM of apoptotic cells after transfection with siRNA_*(LPA1)*_. **D** Quantization of flow detection data in **C** ****p < 0.0001, *n* = 3, as determined by one-way ANOVA.

We investigated the mRNA expression levels of the LPA_1-6_ receptor in cochlear cells by qRT-PCR. LPA_1_ receptor expression was remarkably greater as compared to that of the other receptors (Fig. 8A). To investigate the relationship between the LPA_1_ receptor and LPA’s protective effect on cisplatin-treated HEI-OC1 cells, LPA_1_-silenced cells were generated by transfecting cells with siRNA_(*LPA1)*_, and siRNA_(*control*)_transfected cells served as controls. The siRNA_(*LPA1*)_ mRNA expression level was remarkably decreased in LPA_1_-silenced HEI-OC1 cells in comparison to that in the control cells (Fig. 8B), thus confirming that the LPA_1_ receptor was efficiently silenced. We measured the apoptosis levels after transfection with siRNA_(*LPA1*)_ by flow cytometry. Dead cells and apoptotic cells were marked with PI and Annexin V, respectively [22]. Compared to the siRNA_(*control*)_ group, the siRNA_(*LPA1*)_ group showed a significant increase in apoptotic cell percentage after LPA and cisplatin treatment (Fig. 8C, D). Thus, silencing of the LPA_1_ receptor blocked the protective effect of LPA.

## Discussion

Ototoxic drugs, including aminoglycosides and chemotherapeutic agents, are drugs that induce toxicity in the inner ear’s structure [42, 43]. The potent anticancer agent cisplatin is used to treat various tumors; however, it can also cause ototoxicity and neurotoxicity, which restricts its utility and clinical applications [21, 44]. LPA, which is a bioactive phospholipid that is present at nanomolar to micromolar concentrations in most tissues and biological fluids, participates in several cellular functions such as cell proliferation and migration, neurite retraction, smooth muscle cell contraction, and aggregation of platelets [45–47]. Here, we assessed whether LPA exhibits protective effects on ototoxicity induced by cisplatin. We revealed that a high ROS accumulation in HEI-OC1 cells exposed to cisplatin, accompanied by mitochondrial damage, led to apoptosis and autophagy. Treatment of HEI-OC1 cells with LPA significantly attenuated apoptosis and autophagy induced by cisplatin exposure. Alternatively, LPA_1_ receptor silencing blocked LPA’s protective effect on apoptosis induced by cisplatin.

Cisplatin has been shown to increase ROS levels [32–36]. Moreover, although cisplatin is highly cytotoxic to proliferating cells, it is also toxic to nonproliferative terminally differentiated cochlear hair cells. In the cochlea, cisplatin causes cytotoxicity mainly because of its interference of the cellular redox and inflammatory response to apoptosis [36, 48–50]. In the present study, cisplatin-treated HEI-OC1 cells showed high ROS accumulation, accompanied by mitochondrial damage, resulting in apoptosis, which is consistent with previous reports. Additionally, autophagy may also be involved in ototoxicity caused by cisplatin. At present, it remains unclear whether autophagy activation is destructive or protective [4, 22]. An excess amount of autophagy activation can cause cell death and pathological alterations [51–54]. The induction of autophagy after aminoglycoside-induced ototoxicity can suppress apoptosis and protect cochlear hair cells through the prevention of ROS accumulation [23, 34, 55, 56]. Here, exposure to cisplatin increased the autophagic marker LC3B expression and promoted apoptosis; however, these effects were alleviated by LPA pretreatment, thus indicating that LPA inhibited cisplatin-induced autophagy. These findings agree with the results of a previous study in which cisplatin combined with meclofenamic acid 2 (MA2) reduced cisplatin-induced ROS accumulation, apoptosis, and upregulation of autophagy in HEI-OC1 cells, indicating that MA2 suppresses autophagy induced by cisplatin [4]. Therefore, LPA might have a similar role to that of MA2. Additionally, in the present study, the motion behavior track experiment of zebrafish revealed that cisplatin exposure increased zebrafish activity and their motion speed and distance as compared to those of the control and LPA groups. These findings show that LPA pretreatment remarkably reduced cisplatin-induced motion behavior activity of zebrafish.

LPA and its receptors are involved in several pathophysiological and physiological processes [57–59]. Suckau [60] et al. described LPA_1_, LPA_2_, LPA_4_, and LPA_6_ receptor expression in mouse brain development. LPA_4_ and LPA_6_ show selective expression in HEV endothelial cells (ECs), and LPA_4_ has a critical role in the transmigration of lymphocytes across HEVs in mice [61]. Matsumoto et al. detected LPA_1_ and LPA_4_ expression, but not that of LPA_2_ and LPA_3_, in adult guinea pigs’ outer hair cells [62]. Signals mediated through LPA_4_ enable the formation of the vascular network, which restores normal vascular barrier function in subcutaneous tumor cells [63]. LPA also induces diverse adhesion molecule expression in human umbilical vein endothelial cells through LPA_1_ and LPA_3_ and several downstream effectors such as RhoA-ROCK2-NF-κB [64, 65]. LPA-induced ovarian cancer cell migration requires ezrin-radixin-moesin (ERM) protein activation through LPA_1_ and LPA_2_ [66]. Here, we first determined LPA receptor (LPA_1-6_) expression levels and found that all receptors were expressed in HEI-OC1 cells, except LPA_3_. The LPA_1_ receptor exhibited the highest expression. Moreover, silencing of LPA1 in HEI-OC1 cells by siRNA transfection promoted apoptosis by cisplatin despite LPA pretreatment, thus suggesting that the LPA_1_ receptor is critically involved in LPA’s protective effect on cisplatin-induced apoptosis in HEI-OC1 cells. LPA_1_ couples with G proteins (including G_i/o_, G_q_, and G_12/13_), and it induces various cellular responses through LPA_1_, including Akt, Rho, and MAPK pathway activation that induce physiological responses, which include cytoskeleton changes, cell migration and proliferation, and cell survival [67, 68]. Moreover, the LPA_1_ receptor is expressed in many tissues such as the brain, lungs, heart, spleen, stomach, and skeletal muscle [69, 70]. LPA_1_-deficient mice show defects in brain development and reduced neural precursor cell proliferation [71]. LPA controls cytoskeletal rearrangement and protects against lung injury by increasing the integrity and remodeling of the lung epithelial cell barrier [72, 73]. Our previous studies showed that LPA activates the Rho-ROCK2/p-ERM pathway to regulate the death of hair cells and NIHL by targeting the cytoskeleton [31, 74]. Here, we preliminarily found that LPA_1_ receptor silencing blocked LPA’s protective effect on apoptosis due to cisplatin. Further studies are required to understand the precise regulatory mechanism of the signaling pathway.

In summary, cisplatin treatment induces apoptosis in HEI-OC1 cells; cisplatin and LPA combination can decrease cisplatin-induced accumulation of ROS and HEI-OC1 cell apoptosis. In addition, LPA treatment remarkably suppressed cisplatin-induced autophagy, thereby protecting HEI-OC1 cells. In vivo fluorescence imaging results indicated that cisplatin caused hair cell death and loss, and LPA treatment significantly protected hair cell death and loss. Our findings provide novel evidence that the LPA_1_ receptor has a major involvement in LPA’s protective effect on HEI-OC1 cell apoptosis by cisplatin. This study reports that LPA-LPA_1_ acts as an important regulator of cisplatin ototoxicity, providing insights into the underlying mechanisms and potential therapeutic targets to prevent ototoxicity caused by cisplatin.

## Materials and methods

### Cell culture and drugs

Cisplatin from Hansoh Pharma (Jiangsu, China; #160203) was used. Caspase 3/cleaved caspase 3 (#WL02117), bax (#WL01637), bcl-2 (#WL01556), β-actin (# WL01845) were purchased from Wanleibio (Shenyang, China). LPA was supplied by Cayman Chemical (Ann Arbor, MI; #62215) and diluted in phosphate-buffered saline (PBS) to yield 10 mM stock concentration. The HEI-OC1 cell line was grown in a DMEM high-glucose medium, which was provided by Thermo Fisher Scientific (Waltham, MA); the medium included fetal bovine serum (10%, FBS) from Gibco (Gibco, USA). Culturing was performed at 33 °C under 5% CO_2_ condition. The cell line was used to study the mechanisms by which cisplatin induces ototoxicity at the cellular and molecular levels.

### Flow cytometry-based analysis of apoptosis

The Annexin V kit (RUO; BD Biosciences, Franklin Lakes, NJ) was used to detect cell apoptosis. After collecting and washing the cells twice with PBS, they were resuspended in binding buffer (1×) at 1 × 10^6^ cells/mL concentration. This was followed by incubation of the cell suspension at room temperature in dark with Annexin V and propidium iodide (PI). A BD Biosciences FACS Calibur system was used for data collection, and the collected data were analyzed by FlowJo 7.6 (FlowJo LLC., Oregon, USA).

ROS accumulation was measured by DCFH-DA assay (WLA131; Wanlei Bio, Shenyang, China). After collecting the standing cells, they were washed in PBS and diluted 1:1000 by using the diluent DCFH-DA (dichloro-dihydro-fluorescein diacetate). During incubation at 37 °C, the cells were mixed vigorously every 5 min. Subsequently, the cells were kept at 37 °C for 30 min and mixed rigorously every 5 min. The cells were then subjected to washing for three times with PBS to fully remove the excess amount of DCFH-DA. Finally, the cells in each treatment group were resuspended in 500 µL of PBS for flow cytometry (FCM) analysis.

### Real-time polymerase chain reaction analysis

The RNAeasy Mini kit (#74004; Qiagen, Hilden, Germany) was employed for RNA extraction from HEI-OC1 cells. The RTreagent kit (#330401; Qiagen) was employed for generating cDNA. Next, RT-PCR was performed using Stratagene (M3003; New England Biolabs, Ipswich, MA). Subsequently, PCR was conducted in a 20-μL reaction volume with Luna universal qPCR Mix (10 μL), 1 μL of forward primer, 1 μL of reverse primer, cDNA template (1 μL), and deionized water. By using *GAPDH* as a standard, target gene levels were normalized, and gene expression was determined using the 2^−ΔΔCt^ method. Primer sequences are shown in Table 1.

**Table 1:** List of primer sequences.

Gene name	Sense	Antisense
*LPA1*	5′-GATGTCTCGGCATAGTTC-3′	5′-CCATTAGGGTTCTCGTT-3′
*LPA2*	5′-GCCAG TGCTACTACAACGAGA-3′	5′-ATGCCAGCGAAGAGGTCA-3′
*LPA3*	5′-TTCCACTTTCCCTTCTACTACCTG-3′	5′-TCCACAGCA ATAACCAGCAA-3′
*LPA4*	5′-AAATGAGAAGTGAGACGGCTAT- 3′	5′-CCTCCTGGTCCTGATGGT-3′
*LPA5*	5′-GCCACTCAGAG CCAACG-3′	5′-T GCCCTACAACATCAACC-3′
*LPA6*	5′-T GCCCTACAACATCAACC-3′	5′-ACTTCTTCTAACCGACCA-3′
*GAPDH*	5′-TGGCAAAGTGGAGATTGTTGCC-3′	5′-AAGATGGTGATGGGCTTCCCG-3′

### Transmission electron microscopy

First, trypsinization of HEI-OC1 cells was performed. The cells were then fixed for 1 h in a fixative solution of glutaraldehyde (3%, pH 7.4). The cells were again fixed for 1–2 h in osmic acid (1%) in a 0.1 M sodium cacodylate buffer at pH 7.2. After dehydration with acetone, araldite CY212 was used to embed the cells. The follow-up experimental sample treatment and process were performed by referring to a previous study [4].

### ROS detection and immunofluorescence staining

Mitochondrial ROS levels were detected by DCFH-DA (#WLA131; Wanlei Bio, China). After 24-h exposure to cisplatin (10 μM) with or without 15 μM LPA, the cells were subjected to PBS washing and incubated with 1:1000 diluted DCFH-DA for 30 min. After fixation with 4% paraformaldehyde, a confocal microscope (Olympus, Tokyo, Japan) was used for cell visualization.

DMEM containing 10% FBS was used to culture HEI-OC1 cells. After the treatment, the cells from each group were aspirated with fluid, and the cells were fixed at −20 °C for 15 min with 4% paraformaldehyde. This was followed by washing with 1× PBS (5 min per wash, three times) and immunostaining. Blocking was performed in PBS with Triton X-100 (1%) and BSA (5%) for 1 h. HEI-OC1 cells were incubated at 4 °C overnight with rabbit anti-LC3B antibodies (dilution 1:200; #3868; Cell Signaling Technology, Danvers, MA); the cells were re-incubated for 12 h with secondary antibodies, counterstained for 20 min using DAPI (1:1000; Sigma-Aldrich), and visualized with a confocal microscope (Olympus).

### siRNA transfection

To perform siRNA transfection, siRNA_(*control*)_ and a target-specific siRNA_(*LPA1*)_ were purchased from Thermo Fisher Scientific (siRNA ID: s66922; siRNA_(*LPA1*)_: #4390771; siRNA_(*control*)_: #4390843) to knockdown gene expression. After seeding HEI-OC1 cells onto 6-well plates (at 0.2 × 10^6^ cells/well concentration), the plates were incubated overnight. Cell transfection was performed using 2 mL Opti-MEM (Gibco) with 9 μL of Lipofectamine RNAiMAX provided by Thermo Fisher Scientific and siRNA duplexes (200 nM), and the cells were transfected with siRNA_(*control*)_ (Gibco, USA) by using the same method for 6 h. The cells were then removed, and the medium was then replaced by a complete culture medium. Next, the cells were subjected to 4-h LPA treatment; this was followed by 24-h cisplatin exposure. After cell harvesting, FCM and Western blotting (WB) assay were performed.

### Protein extraction and Immunoblot detection

RIPA lysis buffer supplied by Beyotime Biotechnology (Shanghai, China) was used for total protein extraction from HEI-OC1 cells. In accordance with the instructions of the manufacturer, protein concentrations were estimated with a BCA protein quantification kit from Beyotime Biotechnology. Protein detection and estimation methods were similar to those described in a previous study. The process of protein detection and analysis is the same as that described in reference [4]. β-actin was taken as the loading control. β-Actin and caspase 3/cleaved caspase 3 rabbit monoclonal antibodies (1:1000 dilution) were used as primary antibodies. The membranes were incubated with horseradish peroxidase-conjugated secondary antibodies (1:2000 dilution) for 1 h at room temperature.

### Evaluation of cisplatin’s and LPA’s effects on zebrafish larvae behavior and In vivo imaging

For determining the protective effect of LPA, 5 d *AB WT (wild type) zebrafish larvae were selected as an animal model to investigate LPA’s and cisplatin’s effects in combination and when administered alone on motor behavior. The experimental protocols were approved by the Institutional Animal Care and Use Committee of the Air Force Medical University (Formerly known as the Fourth Military Medical University). Four treatment groups were set in a 24-well plate (round hole), and each treatment group was established with six replicates (one juvenile fish was placed in each hole). The LPA and LPA + cisplatin groups were subjected to pretreatment with LPA for 4 h. The groups were then treated with 10 µM cisplatin for 24 h. No treatment was administered to the control group. EthoVision XT 17.0, a behavioral analysis system, provided by Noldus (Wageningen, Netherlands) was used to record and analyze the motion trajectories of the larvae in light/dark exploration and tapping acoustic stimulation models. The light was switched on/off for 10 min in the light/dark cycle mode, and the procedure was repeated twice. Neuromast cell vitality was evaluated by Zebrafish [Et (krt4: EGFP)] labeled with lateral neuromast hair cells, Et (krt4: EGFP) [75] zebrafish that express green fluorescent protein in sensory hair cells. A large field fluorescence biological stereo microscope (Axio Zoom V16; Zeiss) was connected to a CCD camera (Axiocam506 mono; Zeiss) to record the fluorescence images Et (krt4: EGFP) Zebrafish.

### Statistical analysis

GraphPad Prism 8.0 was employed for statistical analyses. We used Student’s *t* test to compare differences between two groups. Differences among more than two groups were compared by two-way ANOVA followed by Tukey’s multiple comparison test. *P* < 0.05 was assumed to be a significant value [76].

### Supplementary information

Original Data File

## Data Availability

The data supporting the study findings are available following reasonable request from the corresponding author.
