# Supplementary material for: Lysophosphatidic acid exerts protective effects on HEI-OC1 cells against cytotoxicity of cisplatin by decreasing apoptosis, excessive autophagy, and accumulation of ROS
Source: Cell Death Discov. 2023 Nov 15;9:415. doi: 10.1038/s41420-023-01706-5 (PMC10651903; doi:10.1038/s41420-023-01706-5)
Supplement: Supplementary file 1 — Original Data File [file 41420_2023_1706_MOESM1_ESM.pdf]

uncropped images of the original western blots

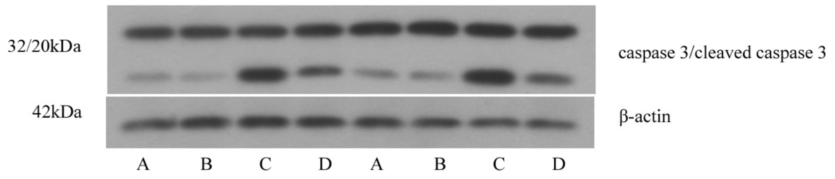

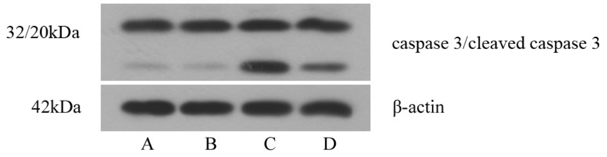

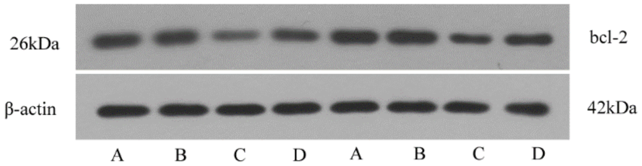

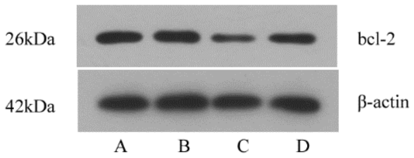

Original image western blot for three repeats

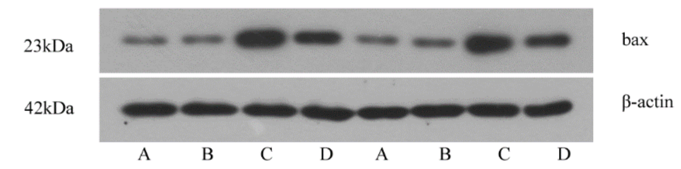

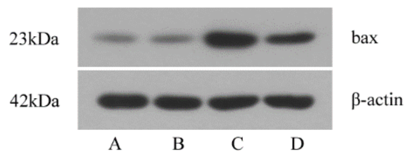

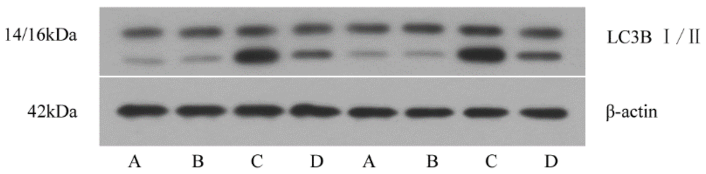

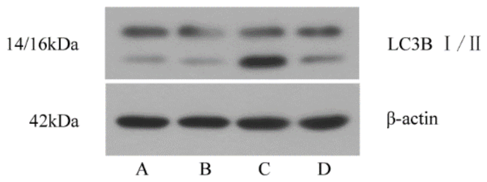

Figure 2(C-I). Original western blot for three repeats

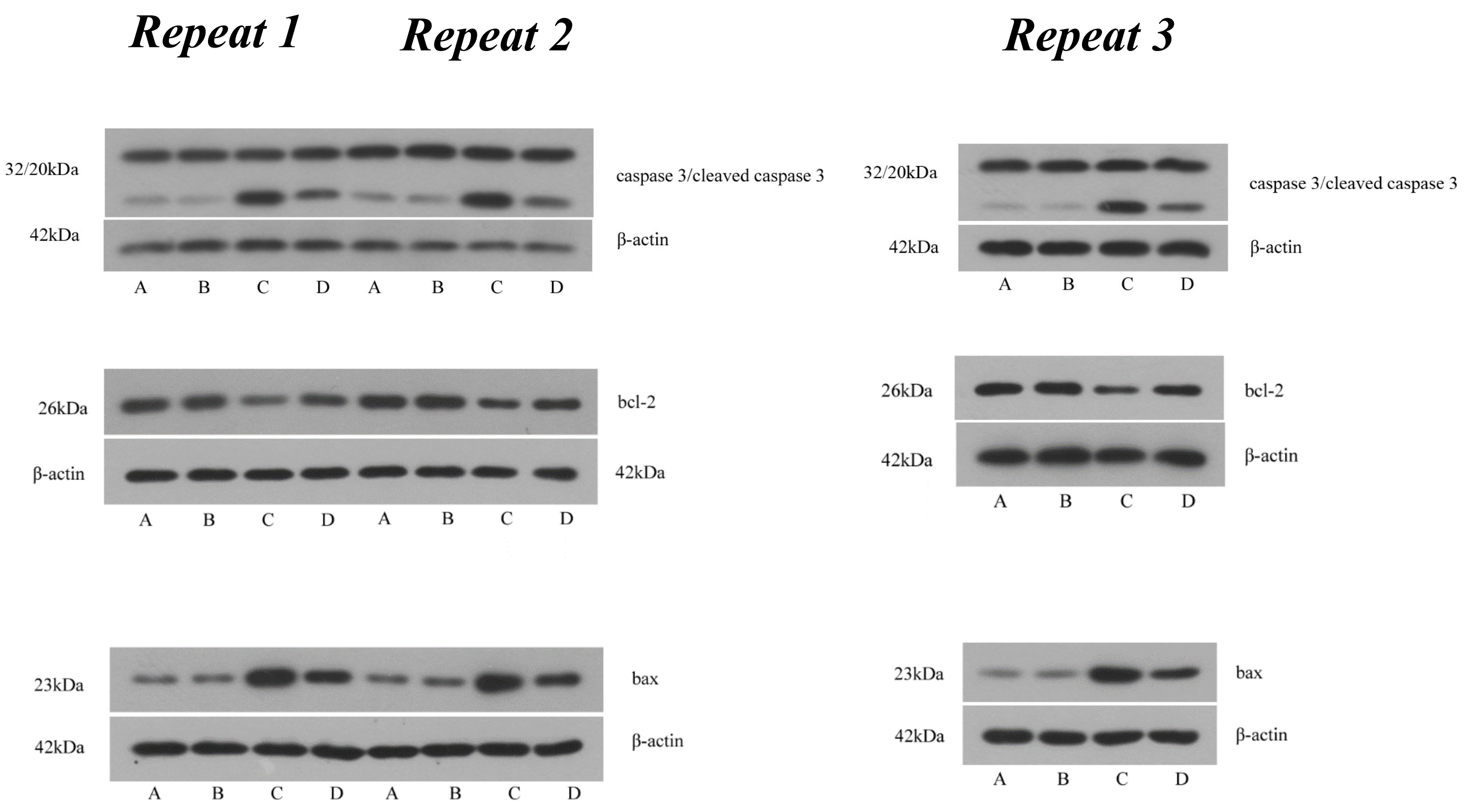

A : control
B : LPA
C : Cisplatin (Cis)
D : LPA+Cis

Figure 5(B-D). Original western blot for three repeats

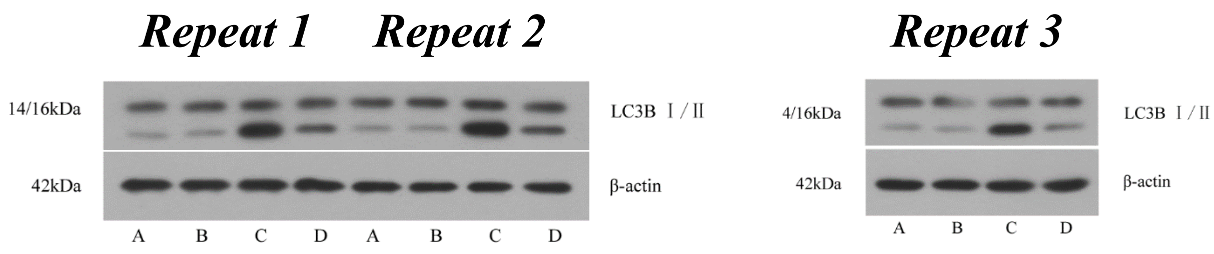

A : control
B : LPA
C : Cisplatin (Cis)
D : LPA+Cis

Reasons why protein Marker cannot be presented on film:

The prestained protein ladder is a mixture of multicolor-stained proteins for use as size standards in protein electrophoresis (SDS-PAGE). We used the prestained protein ladder in our study, which excluded an IgG binding site binding to the primary or secondary antibody used for detection of the target protein. That's why the protein ladder cannot be shown.
